# The role of calcineurin signaling in microcystin-LR triggered neuronal toxicity

**DOI:** 10.1038/srep11271

**Published:** 2015-06-10

**Authors:** Guangyu Li, Wei Yan, Yao Dang, Jing Li, Chunsheng Liu, Jianghua Wang

**Affiliations:** 1 College of Fisheries, Huazhong Agricultural University, Wuhan 430070, China; 2 Institute of Agricultural Quality Standards & Testing Technology, Hubei Academy of Agricultural Sciences, Wuhan 430064, China

## Abstract

Microcystin-LR (MCLR) is a commonly acting potent hepatotoxin and has been pointed out of potentially causing neurotoxicity, but the exact mechanisms of action still remain unclear. Using proteomic analysis, forty-five proteins were identified to be significantly altered in hippocampal neurons of rats treated with MCLR. Among them, Ca^2+^-activated phosphatase calcineurin (CaN) and the nuclear factor of activated T-cells isoform c3 (NFATc3) were up-regulated remarkably. Validation of the changes in CaN and NFATc3 expression by Western blotting demonstrated CaN cleavage and subsequent NFATc3 nuclear translocation were generated, suggesting that exposure to MCLR leads to activation of CaN, which in turn activates NFATc3. Activation of CaN signaling has been reported to result in apoptosis via dephosphorylation of the proapoptotic Bcl-2 family member Bad. In agreement with this, our results revealed that treatment of neurons with the CaN inhibitor FK506 blocked the reduction in Bad dephosphorylation and cytochrome c (cyt c) release triggered by MCLR. Consistent with these biochemical results, we observed a marked decrease in apoptotic and necrotic cell death after MCLR exposure in the presence of FK506, supporting the hypothesis that MCLR appeared to cause neuronal toxicity by activation of CaN and the CaN-mediated mitochondrial apoptotic pathway.

Cyanobacterial blooms constitute a health-risk for human beings via drinking water through the production of a range of hepatotoxins and neurotoxins^1^. Microcystins (MCs) are a diverse group of low molecular weight cyclic heptapeptides produced by a range of distantly related cyanobacteria such as *Microcystis, Anabaena,* and *Planktothrix*^2^,^3^. So far, more than 80 different structural analogues of MCs have been identified. Among these, microcystin-LR (MCLR) is the most abundant and most toxic variant, comprising 46–99.8% of the total MCs in natural waters^4^,^5^.

Earlier studies have confirmed that MCs may accumulate in the brains of aquatic and terrestrial animals and induce neurotoxicity^6^,^7^,^8^,^9^. Although the potential neurotoxicity of MCLR has been proposed, little is known about its molecular basis. We have demonstrated that sub-chronic low dose treatment with MCLR results in neuronal degenerative changes and hyperphosphorylation of cytoskeletal-associated protein tau, causing substantial spatial memory retention deficits in rats^10^. In addition, cognitive impairment and oxidative damage have been observed in rats after intra-hippocampus injection with MCLR^11^. Our previous work has indicated that MCLR inhibits the induction of long-term potential (LTP) in the hippocampus and increases the activity of glycogen synthase kinase-3β (GSK-3β), providing a potential cellular mechanism involved in deficits in cognition caused by MCLR^12^.

MCLR can also be transported into neurons causing cytotoxicity *in vitro*^13^. The toxicity of MCLR at the cellular level manifests as cell blebbing, cellular disruption, loss of membrane integrity and formation of apoptotic bodies, which are all consequences of cytoskeletal disorganization^14^. Previous study employing the neuroendocrine PC12 cell line demonstrated the neurotoxicity of MCLR implicated in neuronal cytoskeletal architecture remodeling and abnormalities in the phosphorylation status of tau and the 27-kDa heat-shock protein (HSP27). It was suggested that this may be caused by neurological protein phosphatase 2 A (PP2A) inhibition and subsequent mitogen-activated protein kinase (MAPK) signaling activation^15^. Investigations in primary murine cerebellar granule neurons showed that MCs exposure induced cytotoxicity, caspase-dependent apoptosis, and microtubule-associated tau protein hyperphosphorylation^16^. In addition, MC-congener-associated PP2A inhibition has been demonstrated in primary murine cerebellar granule neurons. Two hydrophobic MCs, MCLF and MCLW, are more potent than MCLR at inhibiting PPs, and this correlates with their relative potency in causing neurite degeneration^16^,^17^. PPs are responsible for control of many diverse cellular processes, consisting of DNA repair systems, gene expression, protein synthesis and phophorylation/dephosphorylation modification^18^. Perturbation in these pathways, as induced by MCs, can alter the metabolism of cells and lead to necrosis and apoptosis in neurons. MCs can trigger oxidative stress and apoptosis by interaction with the mitochondria of cells^18^,^19^,^20^. It has been suggested that low doses of MCLR lead to apoptosis primarily through the Bid-Bax-Bcl-2 pathway, whereas high doses of MCLR caused apoptosis via a reactive oxygen species pathway by combining the use of standard apoptotic assays with transcriptomics, proteomic technologies, and computer simulations^21^.

However, the molecular mechanisms underlying MCLR toxicity have not been well elucidated regarding to the previous achievements. Global protein information afforded by proteomic analysis provides great value for toxicological studies^22^,^23^, as has been demonstrated in MCLR-induced toxicity studies^10^,^21^,^24^. Studying the signaling pathways and specific MCLR target/interacting proteins in neurons upon exposure to MCLR would not only provide a better understanding of toxicity mechanisms involved with MCs, but also lead to the identification of potential biomarkers that can be used for risk management associated with neurotoxicity of MCs. Thus, we chose two-dimensional electrophoresis (2-DE) combined with matrix-assisted laser-desorption ionization time-of flight (MALDI-TOF) mass spectrometry (MS) analysis to detect global protein profiles of the hippocampal neurons of rats after MCLR exposure.

The main purpose of the current study was to characterize the molecular mechanisms of MCLR-induced neurotoxicity. For that, we analyzed quantitatively changes in protein expression in hippocampal neurons exposed to 0, 0.3, or 3 μM MCLR for 48 h using proteomic analysis. Selected proteins and further proteins associated with these proteins were further confirmed by western blot.

## Methods

### Chemicals

Purified MCLR (purity ≥ 98%) was purchased from Alexis Biochemicals (Lausen, Switzerland). FK506, Modified Dulbecco’s Eagle’s medium (DMEM)/F12 and B27 supplement were obtained from Gibco Invitrogen Corporation (Carlsbad, CA, USA). Hoechst 33258, trypsin, 3-(4,5-dimethylthiazol-2-yl)-2,5-diphenyltetrazolium bromide (MTT) were obtained from Sigma-Aldrich (St. Louis, MO, USA). The reagent kit for determining lactate dehydrogenase (LDH) was purchased from Nanjing Jiancheng Institute of Biological Engineering (Nanjing, China). Primary antibodies anti-calcineurin, anti-NFATc3, anti-Bad, anti-pBad, anti-cytochrome c, anti-GAPDH, anti-COX IV and anti-p62 were obtained from Santa Cruz Biotechnology Inc. (Santa Cruz, CA, USA) and Abcam Limited (Cambridge, UK). All other chemicals utilized in this study were of analytical grade; the chemicals used for electrophoresis were obtained from Amersham Biosciences (Piscataway, NJ, USA).

### Cell culture and exposure

All experimental protocols were conducted in accordance with the *National Institute of Health Guide for the Care and Use of Laboratory Animals.* All experiments conformed to named local guidelines on the ethical use of animals and all efforts were exerted to minimize the number of animals used and their suffering. Primary culture of hippocampal neurons was slightly modified from our previous study^25^. Briefly, hippocampi were carefully isolated from the brain of 1–2 day old Sprague-Dawley (SD) rat pups. The tissues were incubated in 0.125% trypsin for 20 min at 37 *°*C. Neurons were collected and plated at a density of 10^4^−10^5^ per 35 mm^2^ on coverslips or plates pre-coated with 0.1%(w/v) poly-L-lysine in DMEM and F-12 supplement (1:1) with 10% fetal bovine serum (heat-inactivated; Gibco, Carlsbad, USA), 0.5 mM L-glutamine (Sigma, St. Louis, USA), and 1% penicillin-streptomycin. Cells were kept at 37 *°*C in a 5% CO_2_ incubator. After 24 h, the culture medium was changed to DMEM medium containing 2% B27 and 0.5 mM glutamine. Astrocytes were minimized by adding 10 μM cytarabine to the culture medium on day 3. The medium was refreshed every 3 days. After 5–7 days of incubation for cell growth, hippocampal neurons were treated with 0, 0.3, 3 μM MCLR for 48 h. 1 μM FK506 was used to inhibit the activation of CaN. Each treatment was repeated 3 times.

### Proteomic analysis

Proteins were extracted from hippocampal neurons. 2-DE and mass spectrometry analysis were performed as reported previously^10^. Briefly, after protein extraction, the first dimension was carried out using 18 cm pH 4–7 IPG gel strips and 350 μL of sample solution. This pH range allowed proteins with similar isoelectric points (pI) to be separated with high resolution. After isoelectric focusing (IEF), the gels were subjected to a second dimensional electrophoresis on 12.5% polyacrylamide gels. In the second dimension, proteins were separated based on their molecular weight. Finally, the protein spots were visualized via either silver staining or coomassie brilliant blue G-250 staining. After gel image analysis, matrix-assisted laser desorption/ionization time-of-flight (MALDI)-TOF mass spectrometry (MS) was used to identify differentially expressed proteins. The criterion for significant change in protein expression was a difference of at least 2-fold (≥2-fold or ≤0.5-fold) between the treated and control groups. All the 2-DE gels have been run under the same experimental conditions. Each gel is representative of three independent replicates.

### Western blot analysis

Nuclear and cytosolic extracts were prepared by using NE-PER extraction reagent, and mitochondrial fractions were isolated using a mitochondrial isolation kit for cultured cells from Pierce (Pierce, Rockford, IL), according to the manufacturer’s instructions. The rest of the procedures were as described previously by us^10^. Primary antibodies were used as follows: anti-CaN (1:250), anti-NFATc3 (1:500), anti-Bad (1:250), anti-pSer112-Bad (1:250) and anti-Cytc (1:500). Anti-GAPDH (1:2500), anti-COXIV (1:1000) and anti-p62 antibody (1:1000) were used as loading controls. Densitometry was carried out by using IMAGEQUANT TL (Molecular Dynamics).

### CaN activity

The activity of CaN was analyzed using a serine/threonine phosphatase assay kit from Promega (Madison, WI, USA), according to the manufacturer’s instructions. Briefly, the phosphopeptide derived from the RII subunit of cAMP-dependent kinase was used as the substrate. Released free phosphate was detected on a microplate reader by the optical density (OD) at 620 nm, based on the classic malachite green assay. The difference in free phosphate released from reactions in assay buffer and in EGTA buffer indicates specific CaN activity.

### Cell viability, apoptosis and necrosis assay

The integrity of mitochondrial enzymes in viable neurons was evaluated with a colorimetric assay using MTT levels. 48 h after MCLR exposure, the cultures were incubated with MTT solution (0.5 mg/ml) for 4 h at 37 *°*C. The medium was discarded and DMSO was added to solubilize the reaction product formazan by shaking for 10 min. Absorbance at 490 nm was measured with a microplate reader. Cell viability of control groups exposed to no MCLR was defined as 100%. To confirm neuronal death, LDH activity in the medium 48 h after MCLR treatment was determined according to the protocols of the LDH assay kit (Promega, USA).

Fluorescent microscopic studies were performed to distinguish between the apoptotic and necrotic neurons using Hoechst dye (33258) and propidium iodide (PI) labeling. Apoptotic cells were identified on the basis of morphological changes in their nuclear assembly by observing chromatin condensation and fragment staining by the Hoechst dye. Secondary necrotic cells were identified based on positive staining with PI and apoptotic nuclear morphology with Hoechst dye. In each case, at least four microscopic fields were photographed randomly. The experiments were repeated at least twice.

### Statistical analysis

Statistical analyses were performed using SPSS 16.0. Quantitative data were depicted as means with standard errors of the mean (SEM). One-way ANOVA followed by the Duncan multiple group comparison was used to analyze group differences of the data: P values < 0.05 were defined as being statistically significant.

## Results

### Proteome analysis

**Figure 1:**
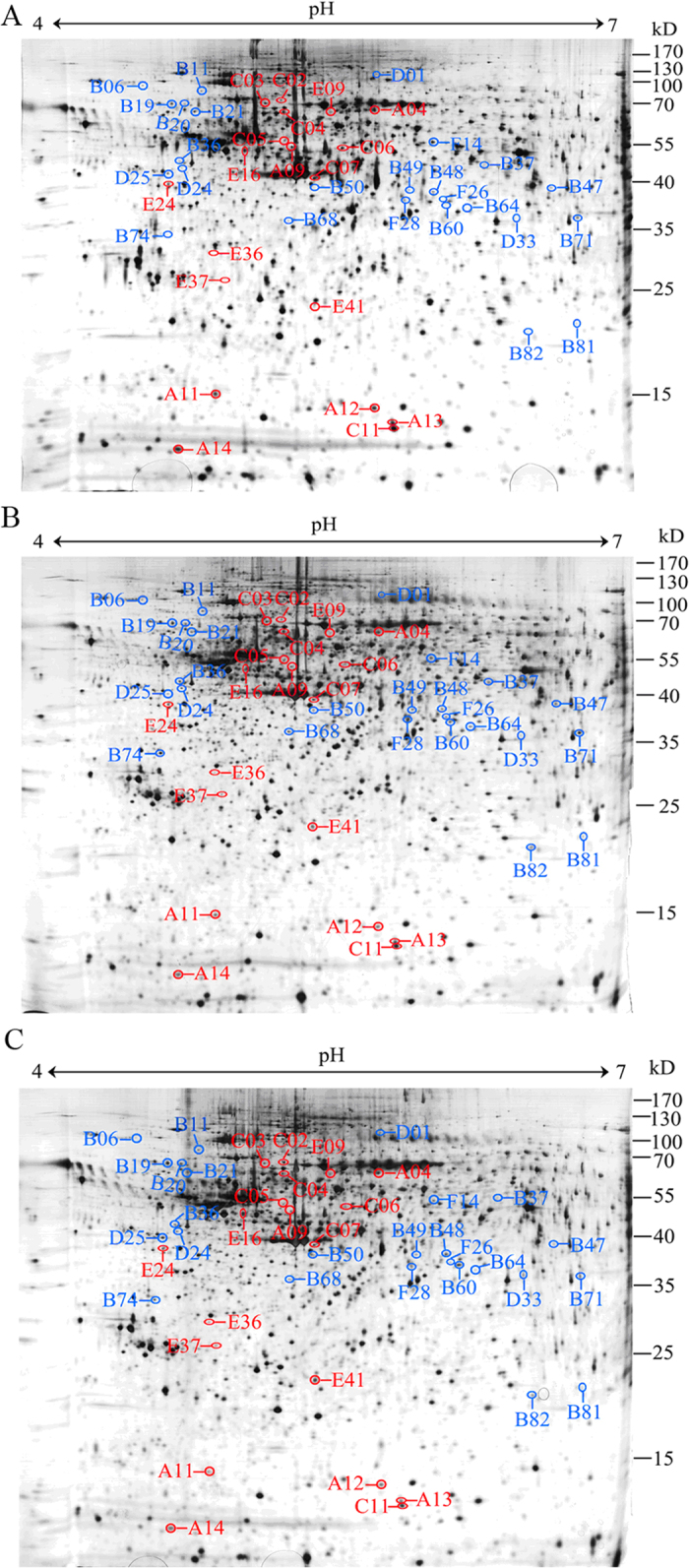
Representative 2-DE gels of the proteins from hippocampal neurons of the control and MCLR-treated groups. (**A**) 2-DE gel image with proteins expressed in the control condition; (**B**) 2-DE gel image with proteins expressed in the 0.3 μM MCLR exposure condition; (**C**) 2-DE gel image with proteins expressed in the 3 μM MCLR exposure condition. The proteins of the samples were separated by 2-DE and visualized by silver staining. Protein spots that were altered by MCLR exposure are labeled with characters. The molecular weights (MW) and pI scales are indicated.

**Figure 2:**
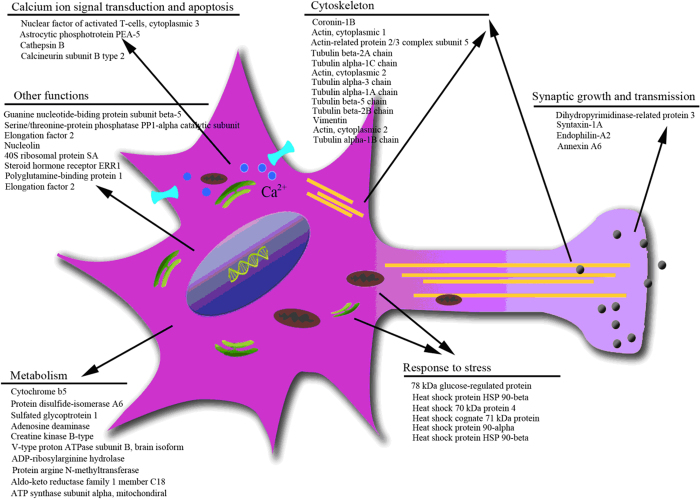
Distribution of the differentially expressed proteins by their function. MCLR-treated neurons showed alterations in proteins involved in calcium ion signal transduction and apoptosis, cytoskeleton, synaptic growth and transmission, metabolism and response to stress. The subcellular components (mitochondria [brown], endoplasmic reticulum [green], cytoskeleton [yellow] and calcium channels [blue]) were affected by MCLR and might contribute to functional deficits related to the cells induced by MCLR exposure.

To further our understanding of the MCLR-induced neurotoxicity and the mechanisms behind it, a proteomic analysis was performed to determine the global effects of MCLR on protein levels of hippocampal neurons. Compared with the gels from the controls, forty-five protein spots were found to have been significantly altered by the effects of MCLR (Fig. 1, Table S1). Of the identified proteins, four proteins (A14, D01, D32 and E41) were characterized as calcium ion signal transduction and apoptosis related proteins. Four proteins (spots B21, D25, E09 and F14) were found to be involved in synaptic growth and transmission. Six proteins (spots B06, B11, B19, B20, B36 and C03) were characterized as response to stress proteins. Thirteen proteins (spots A04, A09, A12, A13, B37, B48, B68, C05, C06, C11, E16, E24 and E37) were found to be cytoskeleton-related proteins, and ten proteins (A11, B47, B50, B81, B82, C04, C07, D24, E36 and F28) were classified as metabolism-related proteins. The other eight proteins were categorized into transcription regulation, protein phosphatase activity, protein biosynthesis, and other functions (Fig. 2).

### MCLR activated CaN/NFATc3 signaling pathway in hippocampal neurons

**Figure 3:**
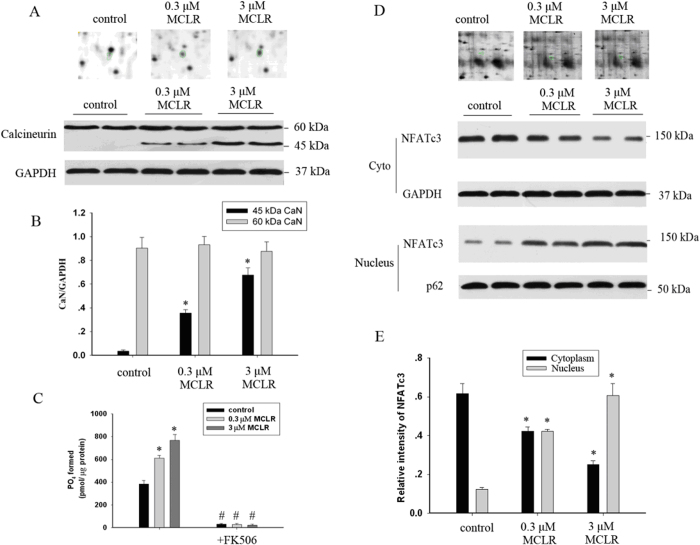
MCLR exposure caused activation of CaN. (**A**) Effect of MCLR on CaN levels. (**B**) Summary data showing cleavage of CaN induced by MCLR. (**C**) Effects of MCLR and FK506 on calcineurin activity. (**D**) Effect of MCLR on the cytosolic and nuclear levels of NFAT3 (E) Summary data showing shuttling of NFATc3 from the cytosol to the nucleus induced by MCLR.

CaN and nuclear factor of activated T-cells, cytoplasmic 3 (NFATc3), were found to be changed after exposure to MCLR as identified by 2-DE analysis. The enlarged representative illustrations in Fig. 3A,D showed a clearincrease in CaN and NFATc3 expression in hippocampal neurons. These changes in protein expression were further validated by Western blot analysis using protein-specific antibodies.

Western blot analysis was performed using a well-characterized antibody that recognizes full-length (60 kDa) and cleaved CaN (45 kDa). As shown in Fig. 3A, cleaved CaN could not be observed in control neurons. Exposure to MCLR resulted in a large increase of the level of cleaved CaN (Fig. 3B). The amount of PO_4_ formed in neurons treated with 0.3 μM MCLR (612.8 ± 29.4 pmol/μg protein) or 3 μM MCLR (768.3 ± 23.6 pmol/μg protein) for 48 h, was significantly higher than in control cells (385.1 ± 31.7 pmol/μg protein), which indicated that CaN is upregulated in treated neurons (Fig. 3C). The CaN inhibitor FK506 almost completely prevented PO_4_ formation in cells untreated or treated with MCLR, a finding that reinforces that we were determining the activity of CaN^26^.

Although originally described in T cells, NFATc3 is now known to participate in the regulation of CaN-mediated transcriptional activity in the nervous system via shuttling from the cytoplasm to the nucleus^27^. Fig. 3D,E showed that MCLR exposure caused a decrease in the cytosolic contents and an increase in the nuclear contents of NFATc3, suggesting that MCLR promotes cytoplasmic/nuclear shuttling of NFATc3.

### FK506 exerted protective efficacy against MCLR-induced neuronal injury

**Figure 4:**
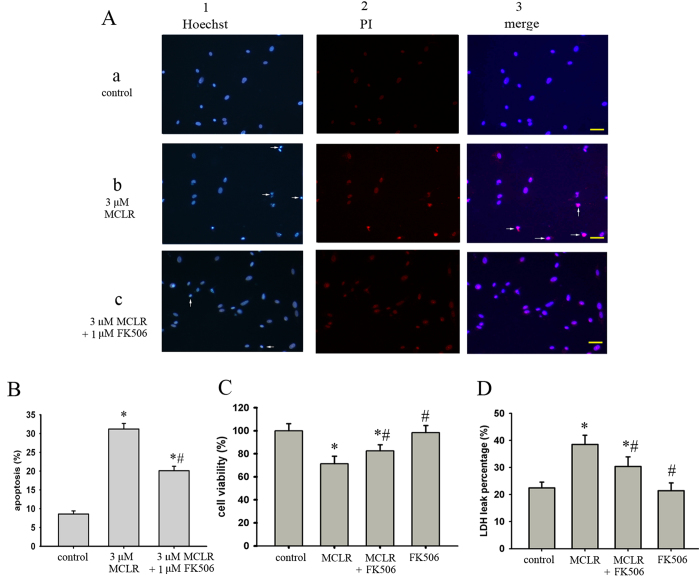
Effects of FK506 on MCLR induced both apoptotic and necrotic cell death in cultured rat cortical neurons. (**A**) Cell apoptosis and necrosis were assessed by Hoechst/PI double staining technique. Normal cells showed uniform blue fluorescence. Apoptotic cells were seen as bright blue fluorescent spots and were shown in Hoechst staining image (arrow). Necrotic nuclei were identified by staining with PI, which showed purple fluorescence in merged image (arrow). Yellow scale bar =50 μm. (**B**) Statistical analysis of apoptotic cell death caused by MCLR. (**C**) Neurons were pretreated with 3 μM MCLR for 48 h followed by 1 h of FK506. Cell viability was determined by the MTT reduction assay and standardized to values from control, which was designated as 100%. (**D**) The extent of cell death was assessed by LDH leakage from the neurons. LDH was measured in medium and cell extracts and expressed as LDH in medium/total (sum of medium and cell extracts). Data were expressed as means±S.E.M. *P < 0.05 compared with control. # P < 0.05 compared with MCLR alone.

We next tested the hypothesis that CaN activation induced by MCLR contributes to neuronal injury. As shown in Fig. 4A, neuronal damage was analyzed by double staining with Hoechst 33258 and PI. MCLR (3 μM) increased the number of apoptotic and necrotic hippocampal neurons as compared to controls, and the pre-treatment of cells with CaN inhibitor (FK506, 1 μM) protected cells from the MCLR-induced apoptosis (Fig. 4A,B, P < 0.05 vs control). As shown in Fig. 4C,D, hippocampal neurons treated with 1 μM FK506 alone showed no significant changes of the cell viability and LDH release. However, 3 μM MCLR decreased the percentage of cell viability to 71.4 ± 6.5% of that of the control (Fig. 4B, n = 4, P < 0.05 *vs* control). 1 μM FK506 tended to prevent the decrease in the cell viability of MCLR exposure but without statistical significance (P > 0.05 vs MCLR alone).

LDH release has been established to correlate positively and linearly with the number of damaged neurons after toxic insults. The membrane integrity after exposure to MCLR was determined using the LDH assay. As shown in Fig. 4D, the percentage of LDH leakage increased from 22.5 ± 2.1% to 38.5 ± 3.4% following treatment with 3 μM MCLR. FK506 prevented LDH release induced by MCLR to 30.4 ± 3.5% (n = 4, P < 0.05 *vs* MCLR alone).

### FK506 prevented Bad activation and mitochondrial cytochrome c release in hippocampal neurons exposed to MCLR

**Figure 5:**
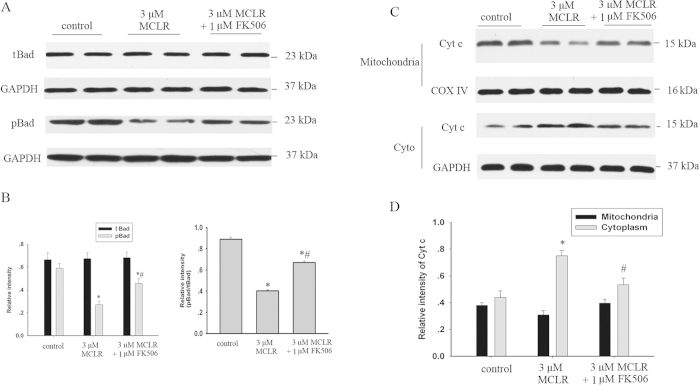
Dephosphorylation of Bad and release of cyt c were induced by MCLR, and these effects were blunted by FK506. (**A**) pBad levels were decreased and the t Bad levels were not changed in hippocampal neurons with exposure of MCLR for 48 h. (**B**) Summary data showing pBad in neurons with non-MCLR and MCLR exposure. The effects of MCLR were blunted by the administration of FK506. (**C**) An increase in cytoplasmic cyt c in neurons with exposure to MCLR. FK506 significantly diminished the amount of cyt c released into the cytoplasm. (**D**) Summary data for mitochondrial and cytoplasmic levels of cyt in neurons. Data were expressed as means±S.E.M. (n = 4 in all groups, * p < 0.05 compared to control, #p < 0.05 compared to MCLR alone).

To determine the mechanism by which CaN activation, triggered by MCLR, led to neuron damage, we examined a known downstream effect of activated CaN, the dephosphorylation of Bad^28^. Phosphorylated Bad (pBad) is sequestered in its inactive form in the cytosol by binding to 14−3−3 protein, whereas dephosphorylated Bad causes cell death by binding to Bcl-x_L_ and Bcl2. To determine the phosphorylation state of Bad, we used a phospho-specific antibody that labels Bad phosphorylated at serine 112 (pBad). To ensure that changes in the relative levels of pBad were not due to differences in the amount of total Bad (tBad), we also determined the levels of this protein using an antibody that recognizes Bad independently of its phosphorylation state. Fig. 5A,B showed that the level of pBad in cultured hippocampal neurons decreased to 45.8 ± 5.4% of the control with 3 μM MCLR treatment, but the level of tBad was unchanged. This decrease was attenuated by pre-incubation of cells with 1 μM FK506, suggesting that MCLR-induced activation of CaN triggered neurons to apoptosis partly via dephosphorylation of Bad at the site Ser^112^.

Dephosphorylated Bad can translocate to the mitochondria and result in cyt c release and apoptosis^29^. To further delineate the sequence of events occurring with MCLR exposure, we examined the cytoplasmic and mitochondrial levels of cyt c. Fig. 5C,D showed cyt c levels to have decreased in the mitochondria and increased in the cytoplasm with MCLR treatment (n = 4, P < 0.05 *vs* control). FK506 blunted significantly the release of cyt c into the cytoplasm in neurons compared to cells receiving MCLR treatment alone (n = 4, P < 0.05).

## Discussion

The findings reported in this study provide information regarding the molecular events of neuronal toxicity upon MCLR exposure. We showed that proteins significantly regulated after exposure to MCLR included calcium ion signal transduction and apoptosis, synaptic growth and transmission, response to stress, metabolic processes or those involved in the formation of the cytoskeleton. The activation of CaN and NFATc3 were identified in our research. We also found that MCLR leads to Bad dephosphorylation, mitochondrial cyt c release and neuron death, and that inhibition of CaN partially blunts Bad dephosphorylation and release which results in prevention for toxicity of MCLR.

CaN is the only Ca^2+^/calmodulin-dependent protein phosphatase in the brain, and it is a major regulator of several key proteins mediating synaptic transmission, neuronal excitability and calcium-dependent apoptosis^30^. Previous studies showed MCLR could increase the intracellular Ca^2+^ concentration and trigger hepatocyte apoptosis^31^,^32^. Consistent with these studies, we found that MCLR exposure could increase intracellular Ca^2+^ concentration in cultured hippocampal neurons (Data not shown). A computational modeling study demonstrated that CaN could be activated with increased Ca^2+^ concentrations^33^. It is thus possible that CaN can remain active in the neurons exposed to MCLR. Due to the fact that MCLR promotes impairment of spatial learning and memory^11^, inhibition of LTP in the hippocampus and loss of neurons^12^, an important role of CaN in MCLR-induced neurotoxicity has been proposed. A constitutive active form of CaN can be formed by cleavage or truncation of the regulatory domains, including the autoinhibitory domain^34^,^35^. In accordance with this, our observations demonstrated that CaN is cleaved and the activity of CaN is increased upon MCLR exposure. The best described substrates of CaN are the NFAT family transcription factors which were first identified in T-cells^36^. Activated CaN induced translocation of NFATc3 to the nucleus, where it activated transcription of target genes^37^. Recently, this pathway was reported to have an important role in the control of the survival/death fate of neurons^35^, and there are increasing data supporting a critical role of CaN/NFATc3 in many events that ultimately lead to cell death^38^,^39^. NFATc3 was decreased in the cytoplasmic fraction but increased in the nuclear fraction after exposure to MCLR for 48 h in our study, suggesting the nuclear translocation of NFATc3 was generated. The activity of NFATc3 can then lead to activation of target genes, which lead to activation or deactivation of cellular reactions^40^.

In order to get more evidence that MCLR exposure regulates CaN signaling, we explored the phosphorylation state of its downstream target, Bad. After being dephosphorylated, Bad translocates to mitochondria and promotes the release of cyt c, leading to the activation of an apoptotic caspase cascade^41^,^42^. Our data showed hippocampal neurons treated with MCLR exhibited lower levels of pBad than untreated neurons, and the effect was prevented by the CaN inhibitor, FK506. Moreover, it was shown that decrease of p-Bad levels was parallel to the increase of cyt c levels in the cytoplasm for neurons treated with MCLR, indicating that the dephosphorylation of Bad triggers cyt c release from mitochondria to cytosol.

We further tested the involvement of CaN signaling in MCLR-induced effects on hippocampal neurons by using FK506, which is known to inhibit the activation of CaN and the apoptosis of cultured neurons^43^,^44^. The mode of action of FK506 has been shown to proceed through the binding of this molecular to specific cytosolic-receptor protein (FKBP12). The FKBP12-FK506 complex binds to CaN to format the ternary complex, which inhibits the activity of CaN. Two widely used inhibitors of CaN are FK506 and cyclosporine A^45^,^46^. In this study we chose FK506 because it can cross the blood-brain barrier. FK506 was found to prevent CaN-mediated dephosphorylation of nitric oxide synthase, leading to neuroprotection^47^. FK506 also inhibited apoptosis by blocking CaN actions to dephosphorylate the proapoptotic Bad, preventing Bad from initiating a cascade leading to cleavage and activation of caspase 9 and apoptosis^48^. According to our data, CaN is only partially responsible for MCLR-mediated neurotoxicity, since FK506 was unable to prevent the apoptotic and necrotic cell death completely. Pretreatment with this drug decreased CaN activity in neurons treated with MCLR, inhibited dephosphorylation of Bad and cyt c release induced by MCLR. However, we cannot rule out the possibility that part of the neuronal toxicity of MCLR is due to non-CaN-mediated mechanisms.

In summary, it is apparent that the activated and cleaved CaN is a key factor involved in the detrimental events following MCLR treatment and the activation of CaN may account for the outcome of neuronal injury.

## Additional Information

**How to cite this article**: Li, G. *et al.* The role of calcineurin signaling in microcystin-LR triggered neuronal toxicity. *Sci. Rep.* **5**, 11271; doi: 10.1038/srep11271 (2015).

## Supplementary Material

Supplementary Information
