# Supplementary material for: The role of calcineurin signaling in microcystin-LR triggered neuronal toxicity
Source: Sci Rep. 2015 Jun 10;5:11271. doi: 10.1038/srep11271 (PMC4462030; doi:10.1038/srep11271)
Supplement: Supplementary Information [file srep11271-s1.pdf]

Supporting Information

The role of calcineurin signaling in microcystin-LR triggered neuronal toxicity

Guangyu Li[1], Wei Yan[2], Yao Dang[1], Jing Li[1], Chunsheng Liu[1], Jianghua Wang[1*]

[1]FisheriesCollege, Huazhong Agricultural University, Wuhan 430070, China

[2]Institute of Agricultural Quality Standards & Testing Technology, HubeiAcademy of Agricultural Sciences, Wuhan 430064, China

***Author for correspondence:**

Jianghua Wang (J. Wang)

College of Fisheries,

Huazhong Agricultural University,

Wuhan 430070, China

Tel: 86-27-87282114

Fax: 86-27-87282114

Email: wangjianghua@mail.hzau.edu.cn

Table S1. A detailed list of protein spots identified by MALDI-TOF/TOF MS from the hippocampal neurons following 0.3 and 3 μM MCLR exposure.

No. on gel	Identification	Fold change[a] 0.3 μM	Fold change[a] 3 μM	Accession no.	Mw(KDa) /pI theoretical	Protein Score	SC[b]	Functional category
	Calcium ion signal transduction and apoptosis							
A14	Astrocytic phosphoprotein PEA-15 (PED)	0.54±0.00	0.50±0.04	gi|61557370	15.08/4.93	115	32%	Apoptosis
D01	nuclear factor of activated T-cells, cytoplasmic 3 (NFATc3)	3.57±0.24	1.97±0.05	gi|157823337	115.25/5.90	129	23%	Transcription regulation
D32	Cathepsin B (Ctsb)	3.25±0.20	5.34±0.30	gi|203647	37.46/5.36	381	28%	negative regulation of cell death;cellular response to mechanical stimulus
E41	Calcineurin subunit B type 2 (CaN)	1.12±0.05	2.08±0.10	gi|11067441	20.29/4.68	175	29%	Calmodulin stimulated protein phosphatase
	synaptic growth and transmission							
B21	Syntaxin-1A (Stx1a)	1.65±0.07	4.46±0.47	gi|58865740	33.06/5.14	61	12%	calcium ion-dependent exocytosis; positive regulation of exocytosis
D25	Endophilin-A2 (Sh3gl1)	4.21±0.13	0.89±0.34	gi|13676846	41.48/5.31	448	39%	Endocytosis
E09	Annexin A6 (Anxa6)	0.43±0.04	0.51±0.13	gi|130502086	76.10/5.39	384	36%	calcium ion transport; membrane-related events along exocytotic and endocytotic pathways
F14	Dihydropyrimidinase-related protein 3 (Dpysl3)	1.63±0.01	2.21±0.05	gi|25742568	62.32/6.04	268	26%	actin crosslink formation; neuron development; SH3 domain binding

	Response to stress							
B06	Heat shock protein HSP 90-beta (Hsp90ab1)	3.57±0.40	1.93±0.32	gi|148747365	83.57/4.97	353	30%	Response to stress; chaperones and folding catalysts
B11	Heat shock 70 kDa protein 4 (Hspa4)	1.98±0.29	2.74±0.41	gi|24025637	94.79/5.13	208	15%	Response to stress; chaperones and folding catalysts
B19	Heat shock cognate 71 kDa protein (Hspa8)	1.63±0.08	2.43±0.26	gi|13242237	71.05/5.37	124	29%	Response to stress; chaperones and folding catalysts
B20	78 kDa glucose-regulated protein (GRP78/Hspa5)	1.77±0.22	3.00±0.48	gi|25742763	72.47/5.07	498	37%	Response to stress; chaperones and folding catalysts
B36	Heat shock protein 90-alpha (Hsp90aa1)	2.00±0.16	1.90±0.42	gi|28467005	85.16/4.93	249	20%	Response to stress; chaperones and folding catalysts
C03	Heat shock protein HSP 90-beta (Hsp90ab1)	0.44±0.03	0.73±0.11	gi|148747365	83.57/4.97	328	30%	Response to stress; chaperones and folding catalysts
	Cytoskeleton							
A04	Coronin-1B (Cor1b)	0.46±0.07	0.57±0.16	gi|9506507	54.43/5.65	345	31%	Cytoskeleton,Actin-binding
A09	Actin, cytoplasmic 1 (Actb)	0.53±0.01	0.49±0.04	gi|13592133	42.05/5.29	580	44%	Structural constituent of cytoskeleton
A12	Actin-related protein 2/3 complex subunit 5 (Aprc5)	0.54±0.01	0.49±0.05	gi|71043638	16.36/5.47	66	30%	Cytoskeleton-actin
A13	Tubulin beta-2A chain (Tbb2a)	0.55±0.01	0.48±0.05	gi|157819845	50.27/4.78	72	8%	Cytoskeleton-tubulin
B37	Tubulin alpha-1C chain (Tuba1c)	2.95±0.58	2.79±0.23	gi|58865558	50.59/4.96	300	28%	cytoskeleton-microtubule
B48	Actin, cytoplasmic 2 (Actg1)	2.26±0.22	4.58±0.15	gi|13592133	42.05/5.29	352	39%	Structural constituent of cytoskeleton
B68	Tubulin alpha-3 chain (Tuba3a)	2.30±0.15	1.94±0.22	gi|399498548	49.95/4.98	320	25%	Cytoskeleton-tubulin

C05	Tubulin alpha-1B chain (Tuba1b)	0.50±0.09	0.86±0.02	gi|112984124	50.80/4.96	141	13%	Cytoskeleton-tubulin
C06	Tubulin alpha-1A chain (Tuba1a)	0.54±0.07	0.43±0.06	gi|11560133	50.78/4.96	111	19%	Cytoskeleton-tubulin
C11	Tubulin beta-5 chain (Tubb5)	0.49±0.01	0.64±0.01	gi|27465535	50.09/4.78	98	13%	Cytoskeleton-tubulin
E16	Tubulin beta-2B chain (Tubb2b)	0.49±0.07	0.41±0.05	gi|110347600	50.37/4.78	192	23%	Cytoskeleton-tubulin
E24	Vimentin (Vim)	0.58±0.00	0.49±0.02	gi|14389299	53.75/5.06	232	42%	Intermediate filament; response to zinc ion; aging;
E37	Actin, cytoplasmic 2 (Actg1)	0.57±0.03	0.27±0.08	gi|13592133	40.05/5.29	295	22%	Cytoskeleton-actin
	Metabolism							
A11	Cytochrome b5 (Cyb5)	0.47±0.01	0.45±0.04	gi|11560046	15.34/4.90	412	49%	Electron transport
B47	Protein disulfide-isomerase A6 (Pdia6)	2.21±0.21	1.23±0.19	gi|52345385	48.54/5.00	372	29%	Isomerase activity
B50	Protein arginine N-methyltransferase 1 (Prmt1)	2.26±0.33	5.60±0.32	gi|13242255	42.12/5.37	160	27%	Methyltransferase activity
B81	Aldo-keto reductase family 1 member C18 (Akr1c18)	3.91±0.20	4.32±0.45	gi|19924035	37.73/5.90	52	21%	Aldo/keto reductase activity
B82	ATP synthase subunit alpha, mitochondrial (Atp5a1)	5.23±0.76	3.77±0.43	gi|40538742	59.83/9.22	413	24%	Energy metabolism
C04	Sulfated glycoprotein 1 (Psap)	0.50±0.15	0.66±0.03	gi|298231223	62.90/5.13	104	8%	sphingolipid metabolic process
C07	Adenosine deaminase (Ada)	0.53±0.03	0.49±0.00	gi|18426812	40.15/5.32	321	35%	Purine metabolism
D24	Creatine kinase B-type (Ckb)	1.92±0.10	2.36±0.25	gi|401461784	42.98/5.39	155	37%	Creatine kinase activity; phosphocreatine

								metabolic process
E36	V-type proton ATPase subunit B, brain isoform (Atp6v1b2)	0.61 ±0.02	0.28 ±0.03	gi|17105370	56.85/5.57	125	19%	ATP metabolic process
F28	ADP-ribosylarginine hydrolase (Adprh)	2.59 ±0.14	1.42 ±0.08	gi|52138626	40.22/5.62	303	37%	ADP-ribosylarginine hydrolase activity
	Other functions							
B49	Guanine nucleotide-binding protein subunit beta-5 (Gnb5)	2.11 ±0.10	1.90 ±0.19	gi|46195741	39.50/5.67	66	23%	G-protein gamma-subunit binding; GTPase activity; chaperone binding
B60	Elongation factor 2 (Eef2)	3.24 ±0.14	1.51 ±0.38	gi|8393296	96.19/6.41	240	17%	Transcription regulation
B64	Serine/threonine-protein phosphatase PP1-alpha catalytic subunit (Ppp1ca)	5.34 ±0.30	3.25 ±0.20	gi|13928710	38.22/5.94	194	46%	protein serine/threonine phosphatase activity
B71	Nucleolin (Nc1)	2.88 ±0.09	2.10 ±0.30	gi|205794	77.14/4.66	172	28%	nucleolar protein of growing eukaryotic cells
B74	40S ribosomal protein SA (Rpsa)	4.39 ±0.26	2.26 ±0.01	gi|8393693	32.91/4.80	116	22%	RNA metabolic process; ribosomal small subunit assembly
C02	Steroid hormone receptor ERR1 (Esrra)	0.36 ±0.03	0.52 ±0.07	gi|158187524	46.06/6.01	29	1%	Cartilage development; regulation of osteoblast differentiation
D33	Polyglutamine-binding protein 1 (Pqbp1)	2.94 ±0.03	1.47 ±0.30	gi|62078643	30.56/5.86	116	35%	regulation of RNA splicing
F26	Elongation factor 2 (Eef2)	5.12 ±0.43	3.00 ±0.25	gi|8393296	96.19/6.43	264	13%	Protein biosynthesis

[a]The fold changes (mean values ± SD, n = 3) are indicated as compared to the controls. Only the fold changes (≥ 2-fold or ≤ 0.5-fold) are shown with their corresponding spot on the other gel. Values > 1 indicate up-regulations, and< 1 down-regulations.

[b]SC indicates the sequence coverage of the protein in percentage obtained by MS/MS identification.
